# Combining *Acmella oleracea* and *Boswellia serrata* extracts: a novel pharmacological approach in inflammatory vestibulodynia

**DOI:** 10.3389/fphar.2024.1508107

**Published:** 2024-12-03

**Authors:** Antimo Fusco, Michela Perrone, Federica Ricciardi, Andrea Maria Morace, Roozbe Bonsale, Milena Melake Teweldemedhin, Emanuele Di Martino, Rebecca Limongelli, Alfonso Papa, Sabatino Maione, Francesca Guida, Livio Luongo

**Affiliations:** ^1^ Department of Experimental Medicine, Division of Pharmacology, University of Campania “Luigi Vanvitelli”, Naples, Italy; ^2^ Department of Pain Management—AO “Ospedale dei Colli”–Monaldi Hospital, Naples, Italy

**Keywords:** microglia, vulvodynia, Acmella oleacea, Boswellia serrata, pain

## Abstract

Vulvodynia is a chronic pain condition that affects the vulvar area, often resulting in significant discomfort and a reduced quality of life. Current treatments for vulvodynia are limited, and there is a need for more effective therapeutic options. Acmella oleracea, known for its spilanthol content, and Boswellia serrata, rich in boswellic acids, have been explored for their potential analgesic properties in pain management. In this study, vulvodynia-like symptoms were induced in female mice using Complete Freund’s adjuvant (CFA). After the induction of symptoms, the mice were treated with a combination of Acmella oleracea and Boswellia serrata extracts (AO + BS). Behavioral pain assessments were conducted to monitor the effects of the treatment. Additionally, biochemical and functional evaluations were performed to measure spinal microgliosis and neuronal overexcitation. The combination of Acmella oleracea and Boswellia serrata (AO + BS) resulted in a significant reduction of vulvar hypersensitivity in mice. Besides alleviating pain, AO + BS therapy also reduced spinal microgliosis and neuronal overexcitation in mice with vulvodynia. The findings suggest that the AO + BS combination has the potential to alleviate vulvodynia associated pain through mechanisms involving the reduction of spinal microgliosis and neuronal overexcitation. These results point to the therapeutic promise of these plant extracts for chronic pain conditions like vulvodynia. The combination of Acmella oleracea and Boswellia serrata shows potential as a treatment for vulvodynia. However, further studies are needed to explore the underlying mechanisms and to optimize the dosage for clinical use.

## Introduction

Vulvodynia is a chronic pain syndrome that significantly impacts on the quality of life of individuals affected (Dagostin Ferraz et al., 2024). Irritation, burning, itching, stinging sensation in the vulvar region and dyspareunia are typical manifestations of the discomfort (Dagostin Ferraz et al., 2024). An approximated 8%–10% of women may experience vulvar pain, though the precise prevalence is likely underestimated due to significant issues of underreporting and misdiagnosis (Bergeron et al., 2020). In fact, despite being relatively common, vulvodynia is inadequately managed with conventional treatments, including tricyclic antidepressants (TCAs), serotonin-norepinephrine reuptake inhibitors (SNRIs) and anticonvulsants, providing a limited relief (Faye and Piraccini, 2023). The precise aetiology of vulvodynia remains unclear, and ongoing research aims to elucidate the factors contributing to its development. Potential mechanisms include nerve injury or irritation and inflammation, which may disrupt the transmission of pain signals from the vulva to the spinal cord. Additionally, an increase in the number and sensitivity of nerve fibres in the vulvar region has been proposed (Faye and Piraccini, 2023). At this regard, we have recently demonstrated that spontaneous or evoked hypersensitivity at the vulvar level, is associated with an overexcitation and neuroinflammation occurring at spinal cord level (Boccella et al., 2024b).

In recent years, animal models have proven invaluable for addressing the limitations of human studies, offering deeper insights into the underlying mechanisms of vulvodynia. Among these, the Complete Freund’s Adjuvant (CFA)-induced vulvodynia model has emerged as a robust tool for exploring the pathophysiology of this condition (Sharma et al., 2018). CFA, composed of heat-killed *Mycobacterium tuberculosis* suspended in mineral oil, is a potent immunological adjuvant commonly used to induce chronic inflammation and pain, providing a valuable method for investigating persistent pain mechanisms (Chakrabarti et al., 2018; McCarson and Fehrenbacher, 2021).

In a recent perspective article, we suggested the possible future molecules potentially useful for the treatment of vulvodynia (Infantino et al., 2024). On these bases, the present study aims to expand this research by evaluating novel compounds as potential therapeutic option for the management of vulvodynia.

Specifically, we assessed the effects of a fixed dose combination of two natural substances *Acmella oleracea* and *Boswellia serrata*, known for their medicinal properties. *Acmella* is recognized for its analgesic capabilities, while *Boswellia* is renowned for its potent anti-inflammatory effects. Their key bioactive compounds, including boswellic acids and spilanthol, act on pathways involving cannabinoid and TRPV1 receptors, supporting their application in chronic inflammatory pain management (Riva et al., 2017; Spinozzi et al., 2022). Here, we tested the effects of *Acmella oleracea* and *Boswellia serrata* combination in CFA-induced vestibulodynia, by using behavioural testing, immunohistochemical and electrophysiological techniques. The results demonstrate the efficacy of the combined treatment in modulating pain sensitivity with significant reduction of spinal biochemical modifications. According to our findings Acmella-Boswellia may represent a natural alternative for the management of pain in vestibulodynia.

## Materials and methods

### Animals

Experiments were performed following the Guidelines for Animal Care and Use of the National Institutes of Health to minimize the number of animals and animal suffering. The experimental protocol was approved by the Ethical Committee of University of Campania “Luigi Vanvitelli”, Italy and the Italian Ministry of Health (Protocol N. 599/2023-PR). Animals were group-housed and maintained under controlled illumination (12 h light/dark cycle) and standard environmental conditions (ambient temperature 20°C–22°C, humidity 55%–60%). Food and water were available *ad libitum*.

### Induction of inflammation

Female mice (C57Ll/6J, 6–8 weeks of age, independent of the estrus cycle) were injected under general anaesthesia (isoflurane, induction 4%, maintenance 1.5% in oxygen) with CFA (5 μL, SigmaAldrich) into the distal vaginal wall by using a pulled glass micropipette to minimize mucosal damages. Control mice received an equivalent volume of 0.9% saline injection. Following intravaginal injections, anaesthesia was withdrawn and mice recovered. Inflammation was produced by administering a single intravaginal injection of CFA every 7 days for a period of 28 days (4 injections in total), while the mice were monitored for a total of 42 days (6 weeks) for the evaluation of pain behaviour.

### Drugs

Acmella oleracea (dry extract 3% alcylamides) and Boswellia serrata (dry extract 25% triterpenic acids) were kindly provided by Sanitas Farmaceutici Srl, Tortona (AL), Italy. Drugs combination was administered in two different formulations:

- ED50 for each component (Acmella 13.01 mg/kg and Boswellia 13.83 mg/kg), dissolved in Kolliphor and saline (2:18), administered exclusively for acute experiments.
- ½ ED50 of the Acmella-Boswellia combination, administered both acutely and chronically. In the chronic treatment, administration began the third week following the CFA injection and continued until the animals were sacrificed. ED50 was calculated as the dose that produced 50% of the maximum observed effect relative to vehicle, using a dose-response curve through non-linear regression analysis (Boccella et al., 2024a). All drugs were administered orally. For acute effects, a single dose was given, while in chronic treatment, the drugs were administered once daily via gavage using calibrated syringes with gastric cannulas to ensure accurate and controlled dosing.

### Experimental design

Animals (n = 5-8 mice per group) were divided into four experimental groups as follows:

1. Control group: mice not subjected to CFA injection and treated with vehicle (saline and Kolliphor solution).
2. CFA-injected mice treated with vehicle: mice subjected to CFA injection and treated with vehicle (saline and Kolliphor solution).
3. CFA-injected mice treated with ED50 Acmella oleracea and Boswellia serrata combination: mice subjected to CFA injection and treated with the ED50 dose of the Acmella and Boswellia combination. This treatment was used only for acute experiments.
4. CFA-injected mice treated with ½ ED50 Acmella oleracea and Boswellia serrata combination: mice subjected to CFA injection and treated with the ½ ED50 dose of the Acmella and Boswellia combination. This treatment was used for both acute and chronic experiments.

**FIGURE 1:**
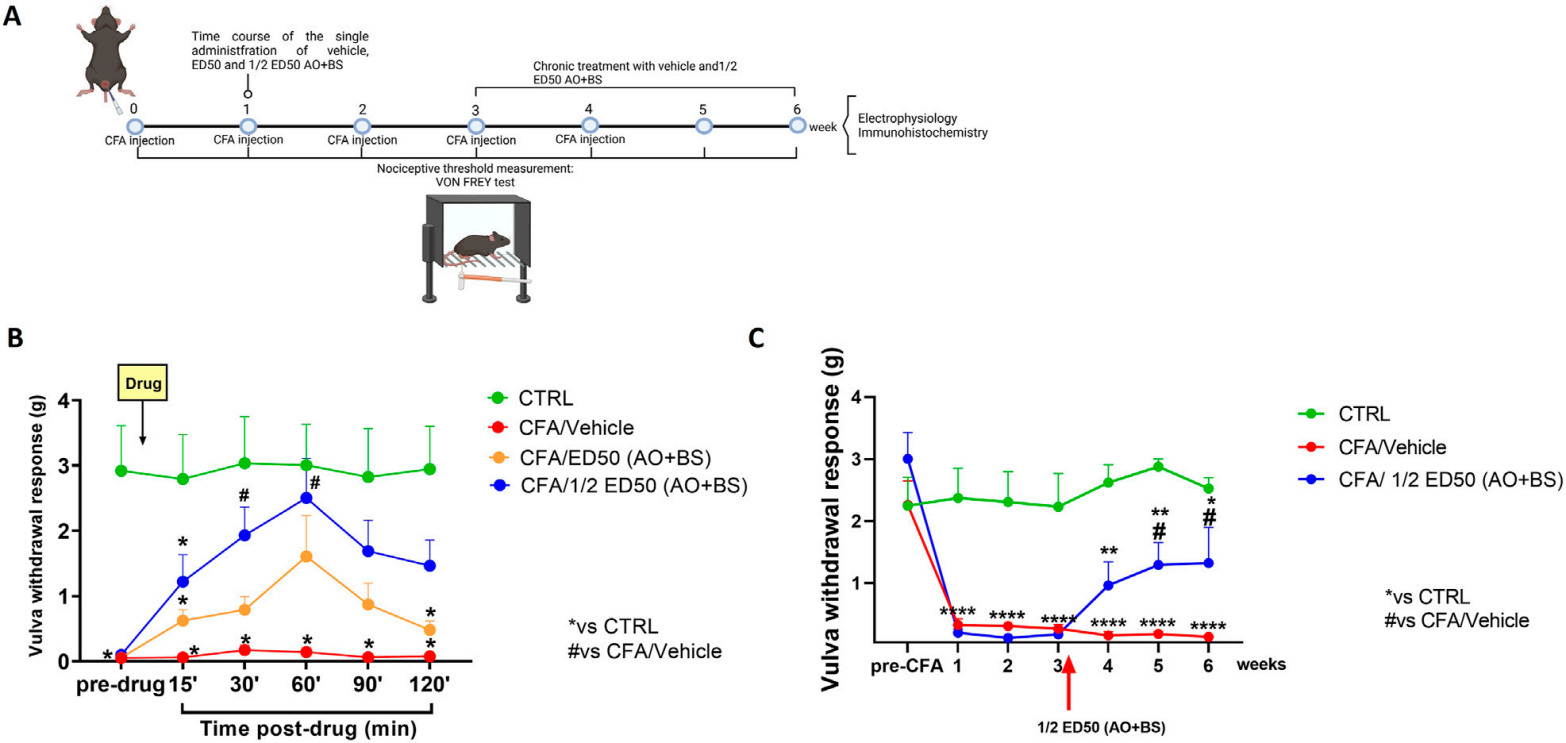
**(A)** Schematic timeline of experimentation (model induction, pharmacological treatments, behavioural testing, electrophysiological and morphological analysis). **(B)** Time-course showing withdrawal responses of the vulva (g) in the Von Frey test at baseline (pre-drug) and for the 120 min following administration of the test substances (ED50 AO + BS and ½ ED50 AO + BS). Data are expressed as mean ± S.E.M. (n = 5 per group). Two-way ANOVA was performed followed by Tukey’s *post hoc* multiple comparison test. P < 0.05 was considered statistically significant. *P < 0.05 vs. CTRL; #P < 0.05 vs. CFA/Veh. **(C)** Time course showing withdrawal responses of the vulva (g) in the Von Frey test at baseline (pre-CFA) and following repeated treatment with ½ ED50 of the AO + BS combination. Data are expressed as mean ± S.E.M. (n = 6–8 mice per group). A two-way ANOVA was performed followed by Dunnett’s multiple comparison test. P < 0.05 was considered statistically significant. *P < 0.05, **P < 0.01, ****P< 0.0001 vs. CTRL; #P < 0.05 vs. CFA/Veh.

Following behavioural tests, mice were divided for electrophysiological evaluations or morphological analysis (Figure 1A). All the experiments were performed by an observer blind to the treatment.

### Von Frey sensitivity testing

Each testing session, preceded by a 3 h-period of habituation to the apparatus, consisted of two measurements1h-intervalled. A calibrated series of Von Frey filaments was applied to the target tissue using the up-down psychophysical method of Dixon (Chaplan et al., 1994), with pressure applied to each filament until it bowed, and held for 2 s. A series of nine von Frey filaments (0.04–4 g) were applied to the vulva (the central, hairless posterior portion) beginning with the 0.6 g filament. Von Frey filaments were disinfected with 70% ethanol between each testing session. The nocifensive endpoint we adopted was a clear reflexive jump or licking of the vulva in response to vulvar stimulation. Data were expressed as the mean of the two different measurements.

### Extracellular recordings of spinal nociceptive specific neurons *in vivo*

*In vivo* extracellular recording has been performed according to our previous studies (Guida et al., 2012; Guida et al., 2015). For *in vivo* single unit extracellular recording, mice were initially anesthetized with Avertin (1.25%). After tracheal cannulation, a catheter was placed into the right external jugular vein to allow continuous infusion of propofol (5–10 mg/kg/h, i.v.). Spinal cord segments L6-S1 were exposed medially by laminectomy, near the dorsal root entry zone, up to a depth of 1 mm. Animals were then secured in a stereotaxic apparatus (David Kopf Instruments, Tujunga, CA, United States) supported by clamps attached to the vertebral processes on either side of the exposure site. The exposed area of the spinal cord was initially framed by agar and then filled with mineral oil. Body temperature was maintained at 37°C with a temperature-controlled heating pad. A glass tungsten filament electrode (3–5 MΩ; FHC Frederick Haer & Co., Bowdoin, ME, United States) was used to record single unit extracellular activity of the Nociceptive Specific (NS) neurons. Neurons were stimulated with a soft bristled brush at the level of the mouse vulva. The mechanical stimulus was applied for 10 s, with 300 s between stimuli in each trial. The recorded signals were amplified and displayed on a digital storage oscilloscope to ensure that the unit under study was unambiguously discriminated throughout the experiment. Signals were also fed into a window discriminator, whose output was processed by an interface CED 1401 (Cambridge Electronic Design Ltd., Milton, United Kingdom) connected to a Pentium III PC. Spike2 software (CED, version 4) was used to create peristimulus rate histograms online and to store and analyze digital records of single unit activity offline. Configuration, shape, and height of the recorded action potentials were monitored and recorded continuously using a window discriminator and Spike2 software for online and offline analysis. The neuronal activity was expressed as spikes/s (Hz). At the end of the experiment, each animal was killed with a lethal dose of urethane (Katter et al., 1996; Notartomaso et al., 2022).

### Immunohistochemistry

Under anesthesia, animals were transcardially perfused first with saline solution, followed by 4% paraformaldehyde in 0.1 M phosphate buffer. After perfusion, the spinal cords were excised and post-fixed for 3 h in the same fixative. The tissues were then cryoprotected for 72 h in 30% sucrose in 0.1 M phosphate buffer, embedded in Optimal Cutting Temperature compound, and frozen. Transverse sections of 20 µm thickness were cut using a cryostat and mounted onto glass slides. The slides were incubated overnight with a primary antibody solution targeting the microglial cell marker Iba-1 (goat anti-ionized calcium binding adapter molecule-1; 1:1,000; Wako Chemicals, Germany). After incubation with the primary antibody, the sections were washed and incubated for 2 h with a secondary antibody (donkey anti-goat Alexa FluorTM 488; 1:1,000; Molecular Probes, United States). Finally, the slides were washed, cover-slipped with Vectashield mounting medium (Vector Laboratories, United States), and prepared for visualization visualized under a Leica fluorescence microscope (Luongo et al., 2013). The staining was than analyzed using the skeletal analysis plugin following the previously published protocol (Morrison et al., 2017).

## Results

### Effects of acute treatment with ED50 and ½ ED50 of the AO + BS combination on mechanical allodynia measured at vulvar level

Before proceeding with the acute administration of the substances under investigation, the nociceptive threshold at the vulvar level of each mouse was assessed for a baseline measurement (pre-drug) using Von Frey filaments. Responses were recorded for the subsequent 120 min following substance administration. At baseline, CTRL-group of mice showed a threshold of 2.918 ± 0.694 g while CFA-injected mice exhibited vulvar allodynia, with a nociceptive threshold ranging between 0.052 and 0.098 g. The administration of the ED50 of the Acmella + Boswellia combination improved the vulvar mechanical nociceptive threshold, although this improvement was not statistically significant, reaching a peak effect at 60 min (1.608 ± 0.629 g, *p* = 0.1615) (Two-way ANOVA; F (5,60) = 3.27 for time, *p* = 0.069; F (2,12) = 13.60 for treatment, *p* = 0.0008; F (10,60) = 2.28 for time x treatment, *p* = 0.024). In contrast, the administration of ½ ED50 of the Acmella + Boswellia combination produced a significant increase in the vulvar mechanical nociceptive threshold, with a peak effect observed at 60 min (2.502 ± 0.607 g, *p* = 0.0371) (Two-way ANOVA; F (5,60) = 3.53 for time, *p* = 0.0351; F (2,12) = 12.73 for treatment, *p* = 0.0011; F (10,60) = 2.68 for time x treatment, *p* = 0.0088) (Figure 1B).

### Effects of chronic treatment with ED50 and ½ ED50 of the AO + BS combination on mechanical allodynia measured at vulvar level

Mice were tested for baseline (pre-CFA) and at each time point after CFA injection (1, 2, 3, and 4 weeks), as well as additional time points at 5 and 6 weeks, for vulvar withdrawal response using Von Frey filaments (Figure 1C). At baseline, all groups of mice showed a nociceptive threshold in the range of 2.245–3.003 g. After CFA injection, we observed a significant decrease in vulvar mechanical withdrawal threshold which persisted until the 6th week (0.130 ± 0.016 g, *p* < 0.0001) (Figure 1B). AO + BS treated mice starting from week three, showed a significant improvement in vulvar mechanical threshold (½ ED50 AO + BS: 1.323 ± 0.575 g, *p* = 0.0341) (Two-way ANOVA; F (6,127) = 9.17 for time *p* < 0.0001, F (2,127) = 71.50 for treatment *p* < 0.0001, F (12,127) = 3.59 for time x treatment *p* = 0.0001) (Figure 1C), compared to vehicle in CFA-injected animals.

### Effect of Acmella + Boswellia combination on the activity of spinal NS neurons in CFA-injected mice

**FIGURE 2:**
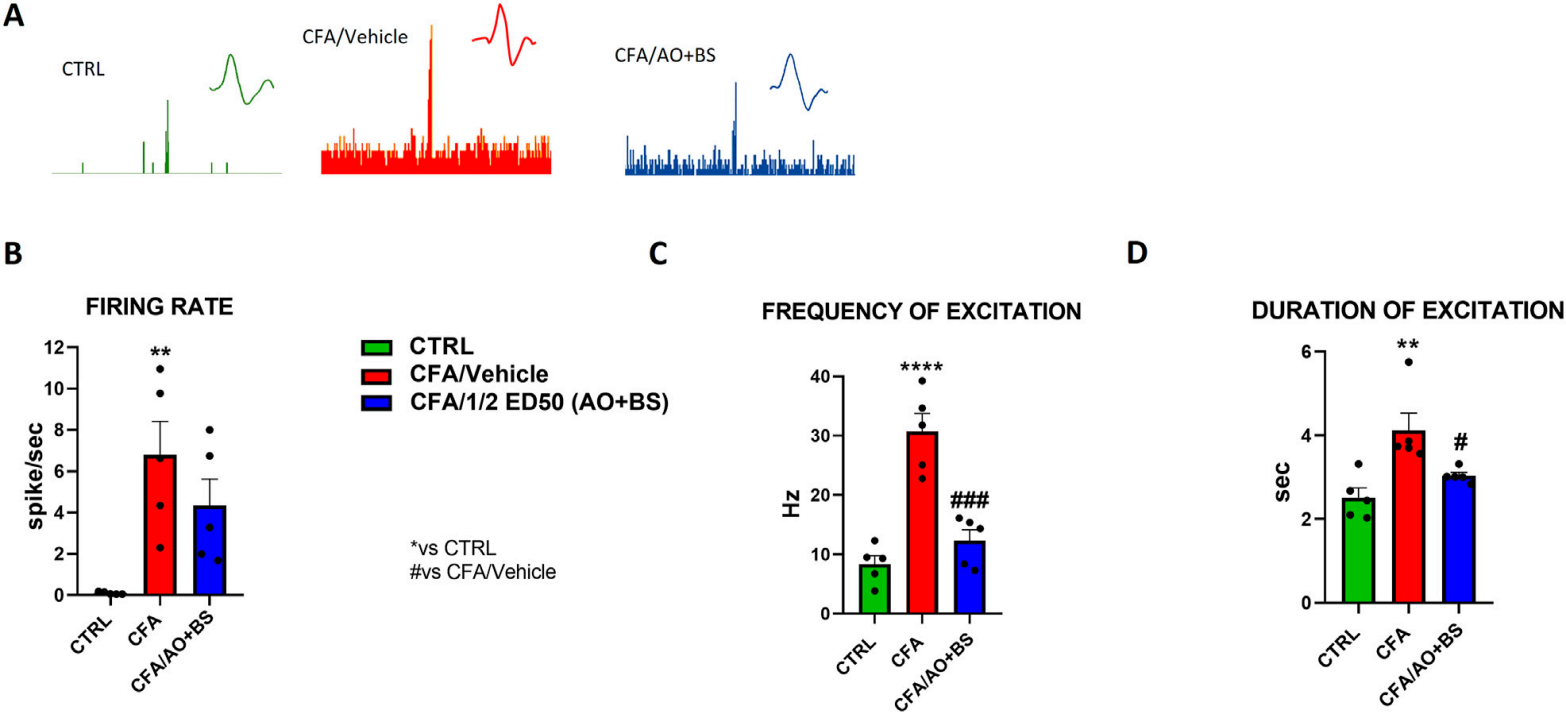
Representative ratemater of NS neurons recorded **(A)** and population data of firing rate **(B)**, frequency of excitation **(C)** and duration of excitation **(D)** in CTRL and CFA mice treated with vehicle or AO + BS Data are expressed as mean ± S.E.M. (n = 5 mice per group). One-way ANOVA followed by Tukey’s multiple comparisons *post hoc* test was performed. P < 0.05 was considered statistically significant. **P < 0.01, ****P< 0.0001 vs. CTRL; #P < 0.05, ###P < 0.001 vs. CFA/Veh.

Electrophysiology experiments through the extracellular single unit technique were performed to investigate the activity of spinal nociceptive neurons between L6 and S2 following CFA vulvar injection with or without Acmella + Boswellia. NS neurons were characterized in the control animals by measuring the mean rate of spontaneous firing 0.100 ± 0.029 spikes/sec of frequency of excitation 8.349 ± 1.449 Hz and duration of excitation 2.514 ± 0.238 s. We observed a significant increase in the spontaneous activity (6.799 ± 1.629 spike/sec, *p* = 0.0048), frequency of excitation (30.713 ± 3.315 Hz, *p* < 0.0001) and duration of excitation (4.122 ± 0.412 s, *p* < 0.0037) after CFA-injection as compared with control mice. However, we found an important decrease of the firing rate in treated group (½ ED50 AO + BS: 4.341 ± 1.288 spike/sec, *p* = 0.3438) (Figures 2A, B). Indeed, one-way ANOVA revealed a significant effect of treatments on the firing rate in CFA-injected animals (F (2, 12) = 8.090, *p* = 0.006). Similarly, we detected a decrease of frequency of excitation (½ ED50 AO + BS: 12.314 ± 1.918 Hz, *p* = 0.0002) (Figures 2A–C) with a significant effect of treatments in CFA-injected animals (F (2, 12) = 29.11, *p* < 0.0001), as well in the duration of excitation (½ ED50 AO + BS: 3.037 ± 0.086 s, *p* = 0.0407) F (2, 12) = 8.857, *p* = 0.0043) (Figures 2A–D).

### Effects of Acmella + Boswellia combination on microglia morphological changes at spinal cord levels

**FIGURE 3:**
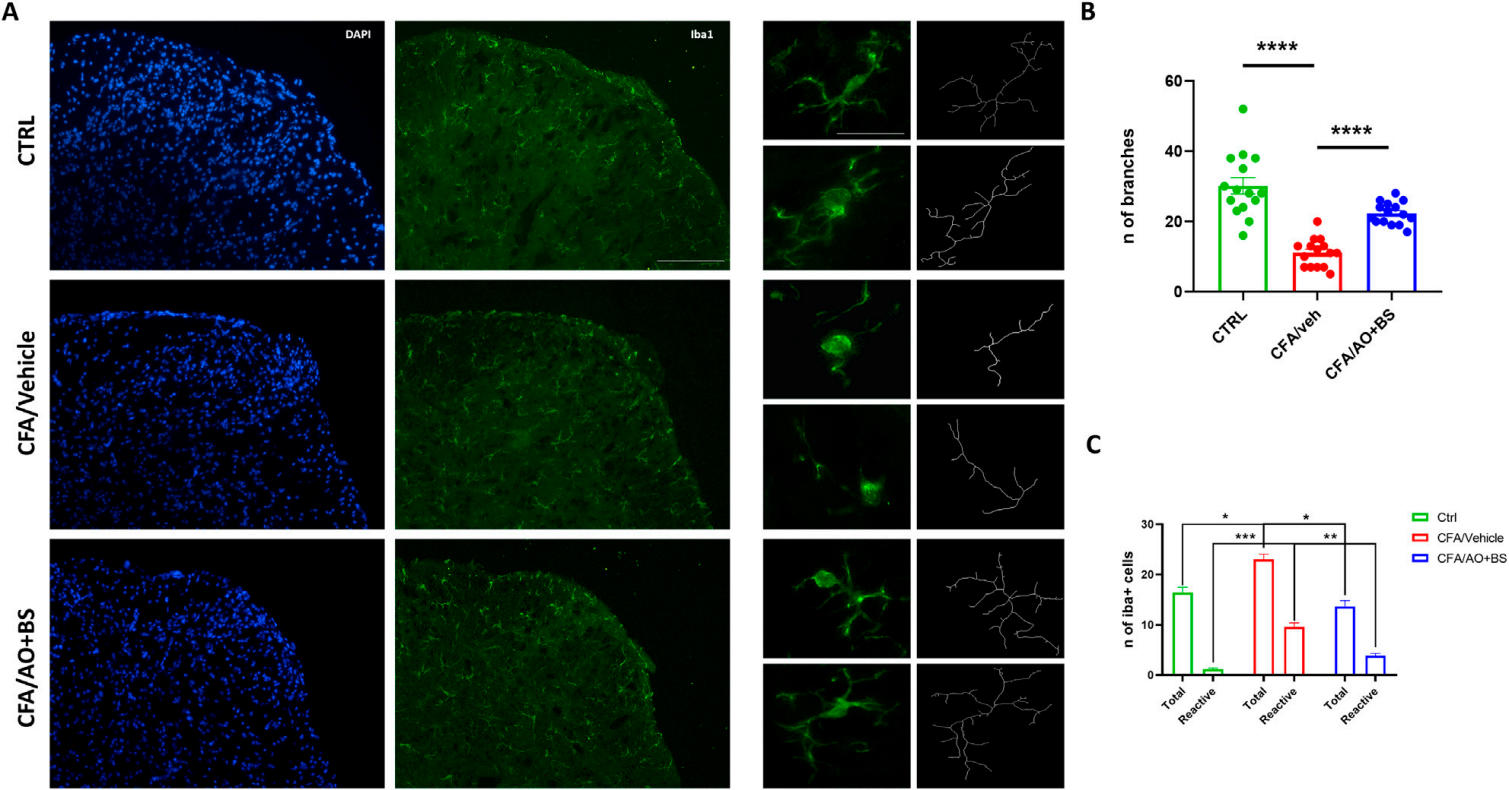
**(A)** Iba1 staining of the L6/S1 segments of the spinal cord (Scale bar = 50 µm) and representation of the skeletal analysis of microglial morphology (Scale bar = 25 µm) in CTRL and CFA mice treated with vehicle or AO + BS. **(B)** Bar graph showing the number of microglial cell branches across the different experimental groups (F (2.24) = 38.49) (n = 3/group). **(C)** Bar graph showing the number of Total and Reactive microglial cell across the different groups (F (2.098, 16.79) = 78.24) (n = 3/group). One-way ANOVA followed by Tukey’s multiple comparisons *post hoc* test was performed. Data are shown as mean ± S.E.M. *P < 0.05, **P < 0.01, ***P < 0.001 and ****P < 0.0001 vs*.* CTRL or CFA/Veh.

Phenotypic changes in microglial cells were assessed through skeletal analysis by quantifying the number of branches at L6/S1 segments of the spinal cord. The results demonstrated a decreased number of branches in microglial cells in CFA/veh group compared to the CTRL group (11.06 ± 1.03 vs. 30.13 ± 2.32, P < 0.0001), indicating a significant alteration in cellular morphology. Interestingly, these morphological changes were reversed after treatment with AO + BS, indeed, the microglial cells in the CFA/AO + BS group exhibited an increased number of branches compared to those in the CFA/vehicle group (22.33 ± 0.802 vs. 30.13 ± 2.32, P < 0.0001). Furthermore, changes in the number of both, total and morphologically reactive cells were observed, indeed the total number of microglia were significantly increased in the CFA/veh group compared to the CTRL group (23.00 ± 1,054 vs. 16.44 ± 1,015, P= 0.0365) as well as the reactive phenotype (9.55 ± 0.867 vs. 1,222 ± 0.222, P = 0.0001). Intriguingly, treatment with AO + BS effectively reduced the number of both total and reactive microglial cells (13.66 ± 1,166 vs. 23.00 ± 1,054; P = 0.0128) (3.88 ± 0.454 vs. 9.55 ± 0.867; P = 0.0063) (Figures 3A–C).

## Discussion

The key finding of this study is the effectiveness of the combined treatment with Acmella and Boswella in reducing pain sensitivity in mice subjected to vulvodynia. Individually, both compounds have been shown encouraging effects in the treatment of inflammatory illnesses and the alleviation of pain (Ammon, 2019). Remarkably, we recently reported that Boswellia and Acmella exert an analgesic effect after peripheral nerve injury in mice, indicating a possible innovative treatment strategy for chronic pain conditions (Boccella et al., 2024a). Particularly, in our previous paper we found a synergistic combination dose through an isobolographic analysis that was performed combining the ED50 values of each substance, to obtain the line of additivity, as previously described (Tallarida, 2000; Miranda et al., 2006).

In this instance, we replicated a vestibulodynia model induced by the CFA injection. CFA causes a potent and persistent inflammatory response, associated with peripheral sensitization, which are similar to the inflammatory conditions observed in human vestibulodynia patients (Sharma et al., 2018; Castro et al., 2022). In AO + BS-treated animals, we observed an important reduction of mechanical vulvar hypersensitivity in established pain condition (3 weeks post CFA-induction). The doses used, based on acute dose response, were effective for the entire course of therapy and observation, by mirroring an antiallodynic effect comparable to that of typical neuropathic painkillers like gabapentin or amitriptyline (Ammon, 2019). Interestingly, we further demonstrated the effectiveness of the AO/BS administration. The most effective combination occurred at half the ED50, whereas using the ED50 doses of both plant extracts resulted in a reduced antiallodynic effect. This outcome, consistent with our previous findings (Boccella et al., 2024a), may be attributed to off-target effects at higher doses. Indeed, at higher doses the N-alkylammides such as spilanthol contained in the Acmella Oleracea could act on several channels, including TRPV1 and K2P potassium channels such as TREK1 (Xu et al., 2019). However, the mechanism of action of this compound still needs further investigation, therefore, at higher doses, it could stimulate other channels inducing a counterbalancing effect. Moreover, also Boswellic acids might act on TRP channels (Liao et al., 2023) or on 15-lipoxygenase-1 (15-LOX) exerting anti-inflammatory and antiallodynic effects (Börner et al., 2023). Finally, the combination mechanism could also involve other pathways that could reveal the synergistic effect. Further studies will be conducted for investigating the molecular pharmacology of this novel phytotherapeutic.

Concurrently with pain relief, AO + BS treatment reduced nociceptive specific neurons overexcitability in the dorsal horn of the spinal cord, which is a hallmark of chronic pain syndromes. In fact, neuroplastic modifications at the spinal areas innervated by nociceptors that supply the distal region of the vagina, may be responsible for abnormal vulvar pain. Indeed, in the long run, CFA causes neuroinflammatory mechanisms which sustain neuronal excitation suggesting a loop-shaped crosstalk of malfunctioning synapses into the spinal cord (Boccella et al., 2024b). Here, AO + BS repeated treatment reduces spinal nociceptive neurons sensitization, as indicated by the normalization of the frequency and duration of neurons excitation. This effect may probably be due to an inhibition of local inflammatory response. We cannot exclude that the main active component of *Acmella Oleracea* (Spilanthol) can directly act on specific potassium channels (K2P, TREK), that are involved in pain chronification (Pereira et al., 2014). Further investigations are needed for better understanding the real mechanisms of action through which *Acmella* exerts analgesic effects.

We recently demonstrated that microglia are indispensable for the development of allodynia in CFA-treated mice (Boccella et al., 2024b). Microglia are dynamic cells, and their morphology is closely tied to their functional state. Healthy microglia cells exhibit homeostatic morphology with several ramifications, including small somas, lengthy processes, and densely arborized branches that survey surrounding area for searching infections or neuronal distress. These cells, following damage or inflammation can be activated and release proinflammatory factors (Paolicelli et al., 2022). Under morphometric point of view, reactive microglia show a retraction their processes and adopt a round amoeboid morphotype. Indeed, a highly branched morphology with numerous fine processes is indicative of microglia in a resting or surveillant state, actively scanning the environment but not yet engaged in a full immune response (Morrison et al., 2017). Immunohistochemical observations indicated that pharmacological treatment with AO + BS also restored the number of branches which appeared decreased in CFA mice. It is reasonable that AO + BS could ameliorate the inflammatory condition and alleviate pain sensitivity by reducing local inflammatory cytokines mostly produced by resident microglia. However, further investigation would be needed to clarify the microglia phenotype and releasing factors. In conclusion, given its joint anti-inflammatory and analgesic properties, the Acmella-Boswellia combination may offer a viable therapy option for vulvodynia, by providing an effective natural therapy as an add on therapy to conventional ones, in chronic pain conditions where a long-term therapy is required. Thus, these results advance our knowledge of prospective vulvodynia-related pain management, however, further research is needed to confirm these benefits and develop standardized treatment protocols tailored to the specific needs of patients.

## Data Availability

The raw data supporting the conclusions of this article will be made available by the authors, without undue reservation.
